# Malaria in the horn of Africa: The ongoing battle against drug resistance

**DOI:** 10.1002/ctm2.1482

**Published:** 2023-11-20

**Authors:** Lucien Platon, Qingfeng Zhang, Jun Cao, Didier Ménard

**Affiliations:** ^1^ Institute of Parasitology and Tropical Diseases UR7292 Dynamics of Host‐Pathogen Interactions Université de Strasbourg Strasbourg France; ^2^ Laboratory of Molecular Parasitology Key Laboratory of Spine and Spinal Cord Injury Repair and Regeneration of Ministry of Education Tongji Hospital Clinical Center for Brain and Spinal Cord Research School of Medicine Tongji University Shanghai China; ^3^ National Health Commission Key Laboratory of Parasitic Disease Control and Prevention Jiangsu Provincial Key Laboratory on Parasite and Vector Control Technology Jiangsu Institute of Parasitic Diseases Wuxi China; ^4^ Center for Global Health School of Public Health Nanjing Medical University Nanjing China; ^5^ Laboratory of Parasitology and Medical Mycology Centre Hospitalier Universitaire Strasbourg Strasbourg France; ^6^ Malaria Parasite Biology and Vaccines Unit Institut Pasteur Université Paris Cité Paris France

Malaria, an infectious disease caused by *Plasmodium* parasites transmitted by *Anopheles* mosquitoes, is a significant global public health concern, having reportedly claimed the lives−50‐60 billion people.^1^ Despite considerable progress having been made, *Plasmodium falciparum*, which is responsible for its severe forms, remains highly prevalent in sub‐Saharan Africa. In 2021, 247 million cases and 619 000 deaths were reported, representing an increase of 6.4% compared with 2019.^2^ Currently, the most pressing concern is the emergence of antimalarial drug resistance, which poses a significant obstacle to effective treatment and control.^2^

A recent publication in the New England Journal of Medicine entitled ‘Increasing Prevalence of Artemisinin‐Resistant HRP2‐Negative Malaria in Eritrea’,^3^ provides worrying evidence of the emergence and spread of parasites in the Horn of Africa with both artemisinin partial resistance (ART‐R) and genome modifications that prevent their detection by rapid diagnostic tests (RDTs), threatening malaria control and elimination campaigns in the region and potentially elsewhere in Africa.

## 1 HISTORICAL PERSPECTIVE: EMERGENCE AND SPREAD OF DRUG RESISTANCE

Malaria control and elimination have a long history and are marked by both success and failure. In the 1950−60s, the Global Malaria Eradication Program (GMEP) initiated by the World Health Organization (WHO) aimed to eradicate malaria using chloroquine (CQ) and dichlorodiphenyltrichloroethane (DDT).^4^ However, these efforts failed, and malaria continues to be a significant challenge in sub‐Saharan Africa. The emergence of CQ‐resistant parasites was a significant contributor to this challenge. Retrospective molecular investigations have confirmed that CQ‐resistant parasites arose from independent geographic locations, including the Thai‐Cambodian border (1957), Venezuela and Colombia (1960), and Papua New Guinea, in the mid‐1970s.^5^ CQ‐resistant parasites were first documented in non‐immune travelers in Kenya and Tanzania in 1978, spreading to inland coastal areas, and from 1983 to Sudan, Uganda, Zambia, and Malawi.

**FIGURE 1:**
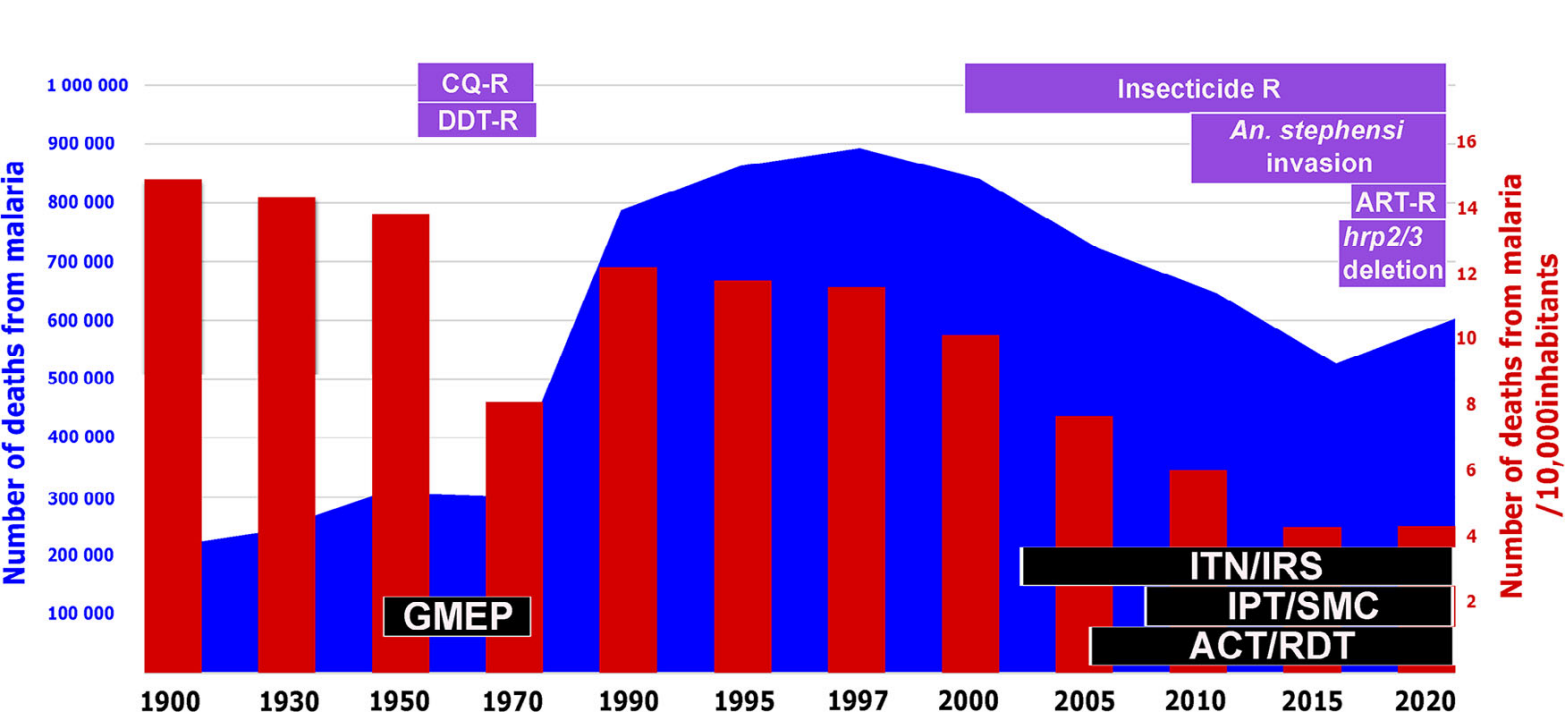
Estimated number of deaths from malaria from 1900 to 2020, strategies implemented to reduce the burden of malaria, and challenges posed by biological threats from vectors and parasites. The curve (blue) and bar charts (red) show the evolution of the estimated number of malaria deaths from 1900 to 2020, and the estimated number of malaria deaths per 10 000 inhabitants during the same period. The strategies implemented to reduce the burden of malaria are shown in the black boxes (GMEP for Global Malaria Eradication Program, ITN for long‐lasting insecticide‐treated bed nets, IRS for indoor residual spraying, IPT for intermittent preventive treatment, SMC for seasonal malaria prevention in children, RDT for rapid diagnostic tests, and artemisinin‐based combination therapy (ACT) for artemisinin‐based combination herapies). The emergence of malaria‐associated biological threats is presented in purple boxes (CQ‐R for chloroquine resistance, DDT‐R for dichlorodiphenyltrichloroethane resistance, Insecticide R for insecticide resistance, and ART‐R for artemisinin partial resistance).

Most countries involved have been left with ongoing malaria burdens, which have been included in malaria control programs since efforts to eradicate malaria have been discontinued. This outcome demonstrated that the attempt to implement a single strategy globally failed and led to the emergence of multidrug resistance and higher mortality rates. Although successful in other regions, the failure of the GMEP in sub‐Saharan Africa emphasized the need for adaptable, region‐specific strategies for malaria control (Figure 1).

## 2 RENEWING THE VISION TO ELIMINATE MALARIA

In the 2000s, WHO launched the Roll Back Malaria Partnership to combat malaria. It brought together stakeholders such as governments, NGOs, and philanthropic foundations to coordinate efforts and resources.^6^ Over the past two decades, several strategies defined by the WHO Global Technical Strategy for Malaria have been implemented to reduce the burden of malaria. Key strategies have included vector control measures such as long‐lasting insecticide‐treated bed nets (ITNs) and indoor residual spraying (IRS), the use of antimalarial drugs as intermittent preventive treatment (IPT) and seasonal prevention of malaria in children (SMC), and the effective management of malaria cases through RDTs and artemisinin‐based combination therapies (ACTs). These strategies, largely supported by the Global Fund, have reduced the incidence and prevalence of malaria and remain essential in the fight against the disease.^2^

## 3 CHALLENGES POSED BY BIOLOGICAL THREATS FROM VECTORS AND PARASITES: A RECURRING SCENARIO

**FIGURE 2:**
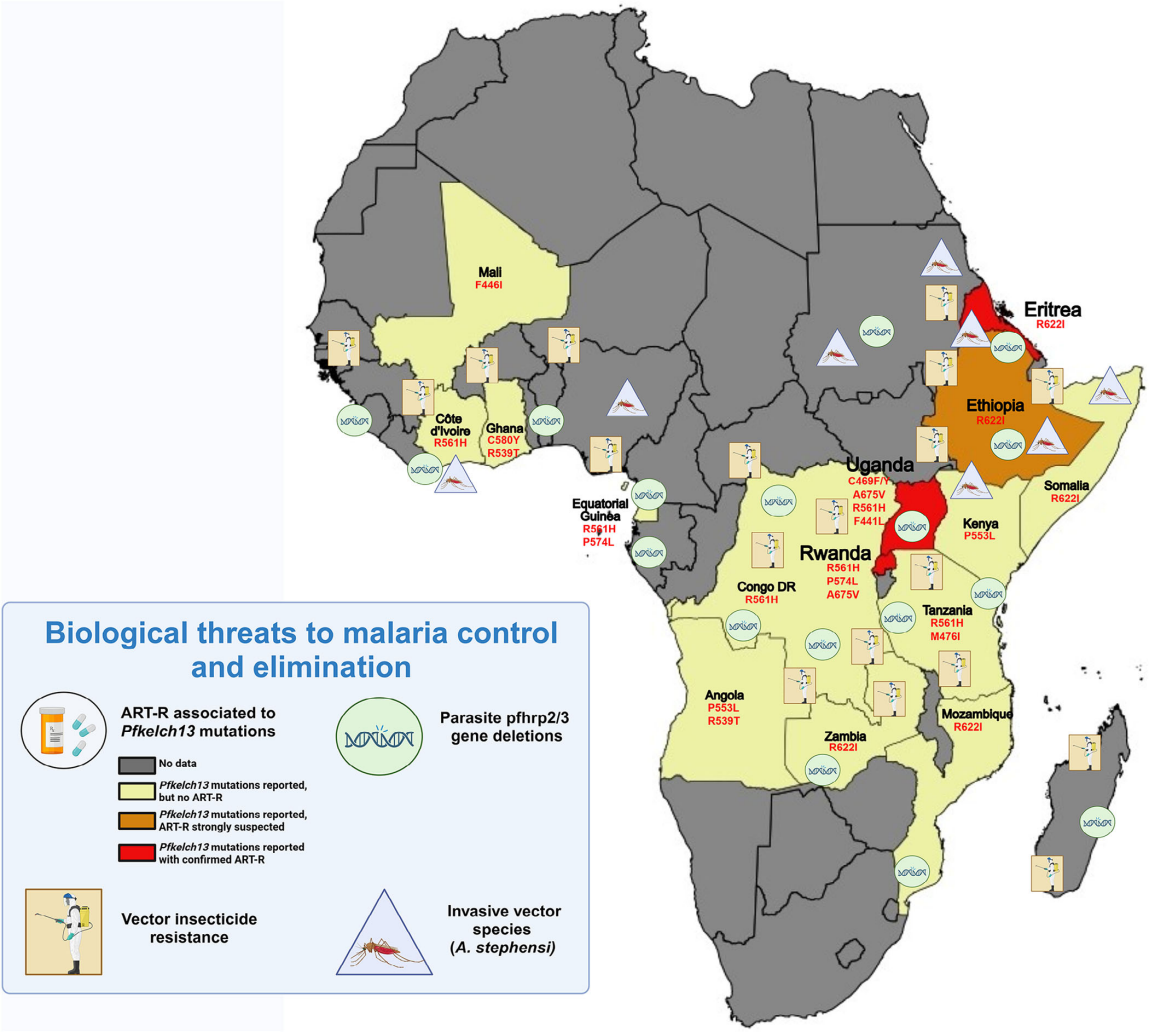
**Geographical distribution of malaria‐associated biological threats in Africa**. The four malaria‐associated biological threats are artemisinin partial resistance (ART‐R) associated with *Pfkelch13* mutants, parasite *pfhrp2/3* gene deletions, vector insecticide resistance and invasive vector species (*An. stephensi*). ART‐R is confirmed in a country if, in a quality‐controlled study using an artemisinin‐based combination therapy (ACT) or an artesunate monotherapy finds more than 5% of patients carry *Pfkelch13* resistance‐validated mutations and have delayed clearance as evidenced by either persistent parasitaemia detected by microscopy at 72 h (± 2 h, i.e., day three) or a parasite clearance half‐life of ≥ 5 h. Validated *Pfkelch13* markers of ART‐R are defined when a statistically significant association between a *Pfkelch13* mutation and clearance half‐life >5 h or day 3 parasitaemia is detected in at least 20 clinical cases and a survival of >1% using the RSA^0−3h^ in at least five individual isolates are detected for a given *Pfkelch13* mutation or a statistically significant difference in the RSA^0−3h^ assay between culture‐adapted recombinant isogenic parasite lines, produced using transfection and gene editing techniques, expressing the variant allele of *Pfkelch13* compared to the wild‐type allele. The list of *Pfkelch13* resistance‐validated mutations can be found at https://www.who.int/news‐room/questions‐and‐answers/item/artemisinin‐resistance (accessed 25 October, 2023).

However, in recent years, there has been a marked increase in the prevalence of vector and parasite threats. Since 2007, ART‐R has been reported in Southeast Asia (SEA), which was later accompanied by resistance to partner drugs. By 2016, the treatment failure rate in some parts of SEA had reached 85%. ART‐R, caused by mutations in the *P. falciparum* parasite gene *kelch13*,^7^ is defined as delayed parasite clearance from the bloodstream in patients treated with ACT. Recently, ART‐R has been firmly established in Central Africa (Rwanda, 2020) and East Africa (Uganda, 2021).^8^ Surveys conducted in various African settings have shown that a significant proportion of *P. falciparum* parasites lack *hrp2/3* genes, especially in the Horn of Africa, which can lead to false‐negative results with HRP2‐based RDTs and potentially compromise patient safety. In addition, since 2010, 85 malaria endemic countries have reported data on insecticide resistance in Anopheles mosquitoes, a major concern given the continued use of pyrethroids in ITNs. Another significant threat is *Anopheles stephensi*, a highly competent vector for both *P. falciparum* and *P. vivax* native to South Asia, which has been reported in several major urban outbreaks in the Horn of Africa (Figure 2).

## 4 AN ALARMING SITUATION IN THE HORN OF AFRICA

Recently, the Horn of Africa has drawn attention as a new hotspot for ART‐R. Clinical drug studies conducted in 2016−19 showed a significant increase in patients with day‐3 positivity after ACT treatment (from .4% in 2016 to 4.2% in 2019) and a substantial rise (from 8.6% in 2016 to 21.0% in 2019) in a new ART‐R variant, *Pfkelch13 622I*, which is accompanied by *Pfhrp2/3* gene deletions in ∼17% of cases, making these parasite strains difficult to detect using HRP2‐based RDTs. Molecular investigations indicated that this phenomenon has been ongoing in western Eritrea for several years.

The results of this study^3^ and two recent reports from Ethiopia^9^, ^10^ highlight the ongoing challenge of drug resistance evolution in the Horn of Africa, which poses a significant threat to global malaria control efforts, and confirm that the strategies being implemented are under threat not only because these ART‐R parasites cannot be detected by RDTs,^10^ but also because malaria vectors in the Horn of Africa are resistant to insecticides and *An. stephensi* can transmit these strains in urban environments.

## 5 CONCLUSION

The fight against malaria in Africa requires both international collaboration and a multifaceted approach that includes strengthening surveillance, investing in research, diversifying treatment options and bolstering vector control efforts.

The past challenges faced by the GMEP serve as reminders that adaptability, region‐specific strategies and ongoing research are essential. The emergence of insecticide‐resistant vectors, spread of *Anopheles stephensi*, and selection of ART‐R/HRP2‐negative parasites in the Horn of Africa emphasizes the need for vigilance and innovation. To overcome these biological threats, we must remain informed and adaptive about our approach, drawing upon the lessons of the past.

## AUTHOR CONTRIBUTIONS

All authors have reviewed, drafted and approved the final version of the manuscript.

## ETHICS STATEMENT

None of the authors have a conflict of interest, financial, or otherwise.
